# Aberrant serum parathyroid hormone, calcium, and phosphorus as risk factors for peritonitis in peritoneal dialysis patients

**DOI:** 10.1038/s41598-020-80938-2

**Published:** 2021-01-13

**Authors:** Chia-Te Liao, Cai-Mei Zheng, Yen-Chung Lin, Mei-Yi Wu, Yuh-Feng Lin, Yung-Ho Hsu, Chih-Cheng Hsu, Mai-Szu Wu

**Affiliations:** 1 Division of Nephrology, Department of Internal Medicine, Shuang Ho Hospital, Taipei Medical University, New Taipei City, Taiwan; 2 Division of Nephrology, Department of Internal Medicine, School of Medicine, College of Medicine, Taipei Medical University, Taipei, Taiwan; 3 TMU-Research Center of Urology and Kidney (TMU-RCUK), Taipei Medical University, Taipei, Taiwan; 4 Division of Nephrology, Department of Internal Medicine, Taipei Medical University Hospital, Taipei Medical University, Taipei City, Taiwan; 5 Institute of Epidemiology and Preventive Medicine, College of Public Health, National Taiwan University, Taipei, Taiwan; 6 Center for Health Policy Research and Development, National Health Research Institutes, Miaoli County, Taiwan; 7 Graduate Institute of Clinical Medicine, College of Medicine, Taipei Medical University, Taipei, Taiwan; 8 Division of Nephrology, Department of Medicine, Tri-Service General Hospital, National Defense Medical Center, Taipei, Taiwan

**Keywords:** Biochemistry, Health care, Medical research, Nephrology, Risk factors, Signs and symptoms

## Abstract

Identifying modifiable risk factors of peritoneal dialysis (PD)-related peritonitis is of clinical importance in patient care. Mineral bone disease (MBD) has been associated with mortality and morbidity in end-stage kidney disease (ESKD) patients. However, its influence on PD related peritonitis due to altered host immunity remains elusive. This study investigated whether abnormal biomarkers of MBD are associated with the development of peritonitis in patients undergoing maintenance PD. We conducted a retrospective observational cohort study, analysing data derived from a nationwide dialysis registry database in Taiwan, from 2005 to 2012. A total of 5750 ESKD patients commencing PD therapy during this period were enrolled and followed up to 60 months or by the end of the study period. The patients were stratified based on their baseline serum parathyroid hormone (PTH) levels, calcium (Ca) levels or phosphorus (P) levels, respectively or in combinations. The primary outcome was the occurrence of first episode of peritonitis, and patient outcomes such as deaths, transfer to haemodialysis or receiving renal transplantation were censored. Peritonitis-free survival and the influence of PTH, Ca, P (individual or in combination) on the peritonitis occurrence were analysed. A total of 5750 PD patients was enrolled. Of them, 1611 patients experienced their first episode of peritonitis during the study period. Patients with low PTH, high Ca or low P levels, respectively or in combination, had the lowest peritonitis-free survival. After adjusting for age, sex and serum albumin levels, we found that the combinations of low PTH levels with either high Ca levels or low/normal P levels were significant risk factors of developing peritonitis. Abnormal mineral bone metabolism in maintenance PD patients with low serum PTH levels, in combination with either high Ca levels or low/normal P levels, could be novel risk factors of PD-related peritonitis.

## Introduction

Globally, peritoneal dialysis (PD) is a major form of kidney replacement therapy (KRT) for patients with end-stage kidney disease (ESKD)^1,2^, offering higher degree of autonomy, flexibility of lifestyle and comparable survival outcomes with hemodialysis^3^. Despite treatment and technique advances, peritonitis remains the major cause of technique failure and mortality in PD patients, hindering its long-term utilization^4–7^. In order to deal with this clinical challenge, worldwide experts from The International Society of Peritoneal Dialysis (ISPD) have published the PD-related peritonitis recommendations, addressing relevant issues such as standardized methods for reporting peritonitis rate, prevention strategies, initial and subsequent management of peritonitis^8^. Notably, several modifiable risk factors of peritonitis have been identified. Of them, medical conditions such as obesity, hypokalemia, hypoalbuminemia, absence of vitamin D supplementation, have been linked to the increased risk of developing peritonitis. Apart from these documented risk factors, recent studies emphasized the importance of host peritoneal immunity in the context of peritonitis occurrence and outcomes^9,10^. Substantial evidence has shown that individual peritoneal immune cell type plays a distinctive role during the process of peritonitis, and that most of them are derived from the circulation under homeostatic process (dialysis exchange) as well as inflammatory process (chemokine-dependent transmigration)^11–13^. Together, these diverse immune cells constitute the systemic and peritoneal immune network, and their dysregulated states under the uremic milieu might result in acquired immune dysfunction in patients with ESKD.

Over the past three decades, parathyroid hormone (PTH) has been recognized with immune-modulatory function, through its corresponding receptor expressed on a wide range of immune cells, such as polymorphonuclear leukocytes and T lymphocytes^14–16^. The impact of PTH on immune effector functions in ESKD patients has been mentioned but the results remain controversial^17,18^. There is very limited data showing that low PTH level is associated with high infection-related mortality in incident dialysis patients^19^. However, it is unclear whether the disordered PTH is related to the occurrence of PD-related peritonitis in incident PD patients. Furthermore, given that serum PTH level is tightly intertwined with serum calcium and phosphorus levels in the context of mineral bone disease in ESKD patients, it would be plausible to investigate the individual and combinational effects of PTH, Ca and P, on the dialysis patient outcomes^20^.

Herein, we hypothesize that PD patients with deranged PTH would exert immune dysregulation to different extent, which may lead to increased or decreased risk of developing peritonitis. Thus, we will examine the relationships between baseline serum PTH (as well as calcium, phosphorus) and the occurrence of first episode of peritonitis in ESKD patients undergoing maintenance PD, based on a nationwide dialysis registry cohort in Taiwan.

## Materials and methods

### Study population

This study was approved by the ethics committee of Taipei Medical University’s joint Institutional Review Board (TMU-JIRB, project N202005075, issued date 23 May 2020) and was carried out in accordance with the principles of the 2013 version of the Declaration of Helsinki 1975. The requirement that patients give informed consent was waived according to the regulation approved by the TMU-JIRB.

In Taiwan, the National Health Insurance (NHI) provides universal health care and covers more than 99% of the Taiwanese population. To comply with the government reimbursement system and the Taiwan Society of Nephrology (TSN) accreditation of dialysis therapy (including PD and HD), all dialysis units are obliged to provide a predefined set of patient-specific health information and laboratory data through a web-based electronic database, and from that the Taiwan Renal Registry Data System (TWRDS) has been established and comprises a nationwide cohort of ESRD patients receiving kidney replacement therapy^21,22^.

In this retrospective, observational study, patients registered with the TWRDS who underwent long-term PD (more than 3 months) from 2005 to 2012 were included in the analysis (n = 14,172). After excluding patients who were age younger than 20 years or older than 90 years (n = 477), has received renal transplantation (n = 34), and had concurrent malignancy (n = 120), and had no PTH, Ca or P data (n = 7791), the final sample for analysis included 5750 patients. (Supplement Fig. 1).

**Figure 1:**
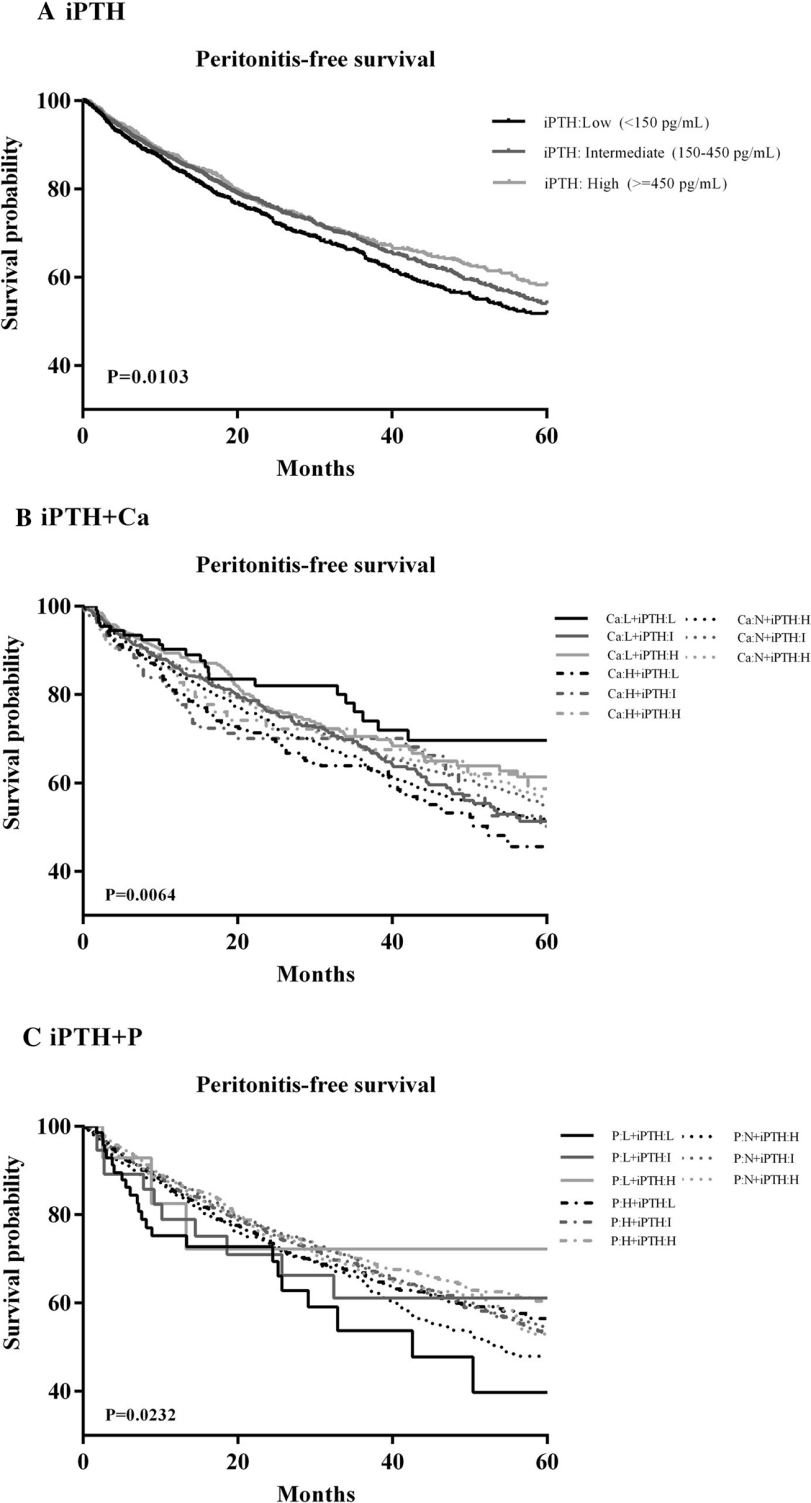
Kaplan–Meier survival analysis demonstrated that patients with (**A**) low intact parathyroid hormone (**B**) low intact parathyroid hormone, combined with low calcium (**C**) low intact parathyroid hormone, combined with low phosphorus, had a worse peritonitis-free survival among all stratified groups, respectively.

### Study outcomes and parameters

All enrolled PD patients were followed until death, transfer to haemodialysis (HD), or kidney transplantation. Patients remaining on long-term PD were censored at the end of 60-month follow-up period. The primary outcome was the occurrence of the first episode of peritonitis diagnosed according to ISPD guideline.

Other patient-specific data such as demographics, primary renal diseases, major comorbid illness were recorded at the time of entering registry. Baseline biochemical data included serum PTH, calcium, phosphorous, sodium, potassium, albumin, hemoglobin, alkaline phosphatase, and parameters related to dialysis adequacy and were recorded at the time of entering registry. The measurements of these laboratory data were specified by the regulations from TWRDS. Serum total calcium was corrected using the following formula: corrected calcium = (0.8 × [normal albumin level – exact albumin level]) + measured serum calcium. The majority of PTH tests in Taiwan were performed using the second-generation PTH Chemiluminescence assay ADVIA Centaur (Siemens Healthcare Diagnostics, Tarrytown, NY) (reference range, 14–72 pg/mL)^22^. Use of antihypertensive drugs and erythropoietin-stimulating agents (ESAs) were also documented in the database.

### Statistical analysis

The data are expressed as means (SD) and frequencies with percentages (%) where appropriate. Group differences were assessed by the one-way analysis of variance for normally distributed continuous variables, the Kruskal–Wallis test for non-normally distributed continuous variables and chi-square test for categorical variables. Kaplan–Meier estimate of survival curves and log-rank tests were performed for descriptive analysis of survival data. The event of interest was the occurrence of peritonitis (first episode) for the analysis of peritonitis-free survival. Modality shift to HD, kidney transplantation and death from any cause were censored observations. The data for a total of 5750 patients were included into univariate and multivariate Cox regression models to determine the influence of PTH/Ca/P on peritonitis. The patients were further stratified three groups according to baseline serum level of PTH, Ca or P, respectively; for PTH, low PTH level: < 150 pg/mL, intermediate PTH level: 150–449 pg/mL, high PTH level: ≧ 450 pg/mL; for corrected Ca, low Ca level: < 8.5 mg/mL, normal Ca level: 8.5–10.2 mg/mL, high Ca level: > 10.2 mg/mL; for P, low P level: < 2.5 mg/mL, normal P level: 2.5–4.5 mg/mL, high P level: > 4.5 mg/mL^23^. Additional analyses for the paired combinations of these parameters will be performed in both survival analysis and Cox regression models.

Two-sided *P* < 0.05 was considered statistically significant. All statistical analyses were performed using the SAS Enterprise Guide software, version 7.1.

## Results

### Patient cohort

The baseline characteristics of the enrolled 5750 patients are shown in Table 1. The mean age was 54.46 ± 14.93 years. The major primary etiologies of ESKD were parenchymal renal disease (25.1%) and systemic disease (33%). The most common comorbidities included type 2 diabetes mellitus (34.28%) and hypertension (22.02%). When stratified into three groups according to their baseline serum PTH level, patients in the low PTH group were more likely to be older and diabetic, and more likely to have higher hemoglobin, higher triglyceride, higher fasting glucose and higher total corrected calcium level. Besides, those with low PTH were more likely to have lower albumin, lower alkaline phosphatase, lower phosphorus, lower normalized protein catabolic rate (nPCR) and normalized protein appearance rate (nPNA). The weekly total Kt/V values across all groups met the current recommended dose (≥ 1.7 per week).

**Table 1:** Baseline characteristics among patients with low, intermediate and high levels of serum intact parathyroid hormone.

Characteristic	Total	iPTH: Low (< 150 pg/mL)	iPTH: Intermediate (150–450 pg/mL)	iPTH: High (≧450 pg/mL)
Total. No (%)	5750	1804 (31%)	2618 (46%)	1328 (23%)
Sex (Male , %)***	2770 (48.17%)	844 (46.8%)	1362 (52%)	564 (42.5%)
Age (Years, mean)***	54.46 (14.93)	57.89 (15.2)	53.76 (14.81)	51.18 (13.84)
Hemoglobin (g/dL)***	10.02 (1.5)	10.13 (1.48)	10.04 (1.51)	9.83 (1.5)
Albumin (g/dL)***	3.61 (0.51)	3.53 (0.54)	3.61 (0.5)	3.7 (0.48)
Aminotransferase (AST) (IU/L)***	24.05 (16.93)	25.39 (18.7)	23.49 (15.34)	23.33 (17.28)
Alkaline phosphatase (IU/L)***	115.81 (94.12)	109.24 (96.57)	110.45 (83.92)	135.51 (106.39)
Cholesterol (mg/dL)**	192.94 (49.54)	191.93 (49.85)	191.95 (49.92)	196.25 (48.24)
Triglyceride(mg/dL)***	155.22 (110.82)	164.19 (107.42)	152.17 (116.59)	149.11 (102.64)
Glucose [fasting] (mg/dL)***	128.78 (71.37)	135.88 (73.54)	128.31 (69.93)	119.92 (70.21)
BUN (pre-dialysis) (mg/dL)***	68.71 (24.09)	64.95 (21.58)	69.26 (23.97)	72.59 (26.62)
Creatinine (mg/dL)***	9.54 (3.15)	8.89 (3.05)	9.63 (3.07)	10.25 (3.25)
Na (meq/L)***	137.28 (4.26)	136.94 (4.27)	137.3 (4.21)	137.67 (4.3)
K (meq/L)***	4.00 (0.73)	3.89 (0.73)	4.03 (0.72)	4.11 (0.75)
Ca (total, corrected) (mg/dL)***	9.17 (0.91)	9.66 (0.93)	9.00 (0.77)	8.86 (0.87)
P (mg/dL)***	4.98 (1.49)	4.63 (1.41)	5.01 (1.45)	5.37 (1.58)
Total KT/V urea	2.17 (0.58)	2.17 (0.59)	2.16 (0.58)	2.19 (0.59)
nPCR (g/kg/day)**	1.06 (0.37)	1.03 (0.28)	1.06 (0.43)	1.08 (0.31)
nPNA***	1.06 (0.37)	1.03 (0.29)	1.06 (0.43)	1.08 (0.3)
Major primary renal diseases (n, %)***				
Parenchymal renal disease	1443 (25.1%)	380 (21.1%)	649 (24.8%)	414 (31.2%)
Systemic disease	1899 (33%)	590 (32.7%)	951 (36.3%)	358 (27%)
Obstructive uropathy and Urinary system	37 (0.6%)	14 (0.8%)	13 (0.5%)	10 (0.8%)
Renovascular problems	3 (0.1%)	2 (0.1%)	1 (0%)	0 (0%)
Genetic disease	67 (1.2%)	18 (1%)	30 (1.2%)	19 (1.4%)
Other	2301 (40%)	800 (44.4%)	974 (37.2%)	527 (39.7%)
**Major comorbid illness (n, %)**				
Diabetes mellitus***	1971 (34.28%)	723 (40.1%)	950 (36.3%)	298 (22.4%)
Hypertension	1266 (22.02%)	384 (37.1%)	589 (40%)	293 (38.8%)
Coronary artery disease*	85 (1.48%)	35 (3.4%)	39 (2.7%)	11 (1.5%)
Cerebrovascular disease**	51 (0.89%)	27 (2.6%)	19 (1.3%)	5 (0.7%)
Liver disease	101 (1.76%)	41 (4%)	39 (2.7%)	21 (2.8%)
**Medications (n, %)**				
Erythropoietin-stimulating agents (ESAs)	3471 (60.37%)	1071 (83.7%)	1578 (85.1%)	822 (87.2%)
Antihypertensive drugs**	2698 (46.92%)	819 (64%)	1277 (68.8%)	602 (63.8%)

BUN, blood urea nitrogen; Na, sodium; K, potassium; Ca, calcium; P, phosphorus; nPCR, normalized protein catabolic rate; nPNA, normalized protein appearance rate; Statistical differences among three groups were shown.

*p < 0.05, **p < 0.01, ***p < 0.001.

### Differential roles of serum PTH, Ca and P on peritonitis-free survival

To explore the potential impact of aberrant serum PTH/calcium/phosphorus levels (individual or combined effects) on the occurrence of PD-related peritonitis, we longitudinally followed up these patients for 60 months and analyzed their peritonitis-free survivals for comparisons. Of total 5750 patients, 1611 patients experienced their first episode of PD-related peritonitis during the study period. 586 patients remained on PD therapy by the end of 60-month follow-up. The Kaplan–Meier estimate of the survival curves and the log-rank tests showed that patients with low serum PTH (*P* < 0.01), high serum Ca (*P* < 0.01), low serum P (*P* < 0.05) had significantly worse peritonitis-free survivals, compared to the other groups, respectively (Fig. 1 and Supplemental Fig. 2). Further analysis of potential synergistic effects of these three biochemical markers demonstrated that low PTH/high Ca, low PTH/low P or high Ca/low P were associated with the worst peritonitis-free survival outcomes (Fig. 1 and Supplemental Fig. 2).

**Figure 2:**
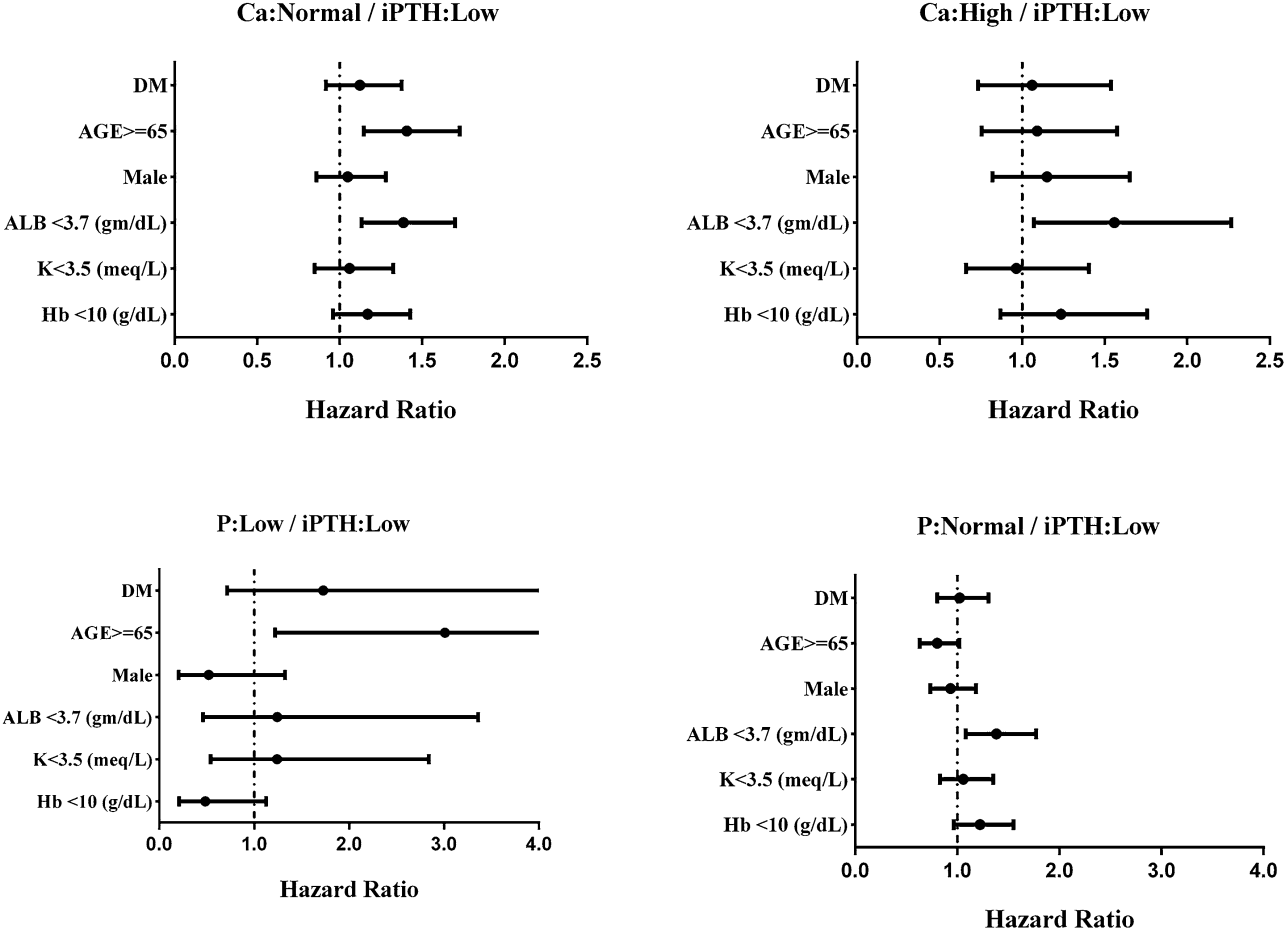
Subgroup analysis revealed that patients with age more than 65-year-old have significantly higher risk for peritonitis occurrence than those less that 65-year-old in the subgroups of “Ca: Normal/iPTH: Low” and “P: Low/iPTH: Low”. Patients with hypoalbuminemia (serum albumin < 3.7 g/dL) have significantly higher risk for peritonitis occurrence than those without hypoalbuminemia in the subgroups of “Ca: Normal/iPTH: Low”, “Ca: High/iPTH: Low” and “P: Normal/iPTH: Low”.

### Aberrant serum PTH, Ca and P levels are risk factors for the occurrence of first episode of PD-related peritonitis

To further examine whether abnormal serum PTH/Ca/P levels at baseline are potential risk factors for the development of PD-related peritonitis, we carried out univariate and multivariate Cox proportional regression models based on the time of PD start to the first episode of peritonitis. As shown in Table 2A-C, increased age and hypoalbuminemia (serum albumin < 3.7 g/dL *versus* ≧ 3.7 g/dL) were two significant risk factors for the development of first episode of peritonitis in the multivariable model, but low serum PTH alone was not a risk factor (Table 2A). Similarly, high serum Ca or low serum P alone was not significant risk factor after adjusting for age, sex and serum albumin (Suppl. Table 1A,B). However, when analyzing data based on the paired combinations, low PTH + high Ca (HR = 1.269) (Table 2B) as well as low PTH + normal P (HR = 1.187) or low PTH + low P (HR = 1.541) (Table 2C) were significantly associated with the early experience of PD-related peritonitis in this cohort. Notably, any combination of serum Ca and P had no influence in this regard (Suppl. Table 1C).

**Table 2:** Crude and multivariable Cox regression analysis of (A) intact parathyroid hormone (iPTH) levels as risk factor for peritonitis occurrence, (B) combined intact parathyroid hormone (iPTH) and calcium (Ca) levels as risk factor for peritonitis occurrence, (C) combined intact parathyroid hormone (iPTH) and phosphorus (P) levels as risk factor for peritonitis occurrence.

Characteristic	Crude			Multivariable				
	β	HR	95% CI	β	HR		95% CI	
**A**								
Age (year)	0.002	1.010*	1.006–1.013	0.007	1.007*		1.003–1.010	
Sex (Male)	− 0.024	0.976	0.885–1.076	− 0.025	0.976		0.884–1.076	
Albumin								
< 3.7 (gm/dl)	0.358	1.430*	1.296–.579	0.310	1.363*		1.233–1.508	
≧3.7 (gm/dl)	–	1	–	–	1		–	
iPTH								
Low < 150 (pg/ml)	0.121	1.128*	1.009–1.262	0.081	1.084		0.969–1.214	
Intermediate 150–450 (pg/ml)	–	1	–	–	1		–	
High ≧450 (pg/ml)	− 0.078	0.925	0.815–1.050	− 0.045	0.956		0.841–1.086	
**B**								
Age	0.010	1.010*	1.006–1.013	0.007	1.007*		1.003–1.010	
Sex (Male)	− 0.024	0.976	0.885–1.076	− 0.016	0.984		0.891–1.086	
Albumin								
< 3.7 (gm/dl)	0.358	1.430*	1.296–1.579	0.307	1.359*		1.228–1.504	
≧3.7 (gm/dl)	–	1	–	–	1		–	
iPTH + Ca								
iPTH:I + Ca:N	–	1	–	–	1		–	
iPTH:L + Ca:L	− 0.365	0.694	0.453–1.063	− 0.327	0.721		0.471–1.105	
iPTH:I + Ca:L	0.058	1.060	0.886–1.268	0.096	1.101		0.919–1.318	
iPTH:H + Ca:L	− 0.143	0.867	0.697–1.078	− 0.073	0.93		0.747–1.157	
iPTH:L + Ca:N	0.136	1.145*	1.004–1.306	0.105	1.11		0.973–1.267	
iPTH:H + Ca:N	− 0.023	0.977	0.838–1.139	0.005	1.005		0.862–1.173	
iPTH:L + Ca:H	0.296	1.344*	1.105–1.635	0.238	1.269*		1.042–1.546	
iPTH:I + Ca:H	0.182	1.200	0.876–1.643	0.166	1.18		0.861–1.618	
iPTH:H + Ca:H	− 0.006	0.994	0.655–1.509	0.007	1.007		0.663–1.531	
**C**								
Age (Year)	0.010	1.010*	1.006–1.013	0.007	1.007*		1.003–1.011	
Sex (Male)	− 0.024	0.976	0.885–1.076	− 0.024	0.976		0.885–1.077	
Albumin								
< 3.7 (gm/dl)	0.358	1.430*	1.296–1.579	0.307	1.360*		1.229–1.505	
≧3.7 (gm/dl)	–	1	–	–	1		–	
iPTH + P								
iPTH:I + P:N	–	1	–	–	1		–	
iPTH:L + P:L	0.526	1.692*	1.106–2.588	0.432	1.541*		1.007–2.359	
iPTH:I + P:L	0.162	1.176	0.644–2.147	0.141	1.152		0.631–2.104	
iPTH:H + P:L	0.164	1.178	0.378–3.676	0.082	1.086		0.348–3.389	
iPTH:L + P:N	0.202	1.224*	1.037–1.444	0.172	1.187*		1.006–1.401	
iPTH:H + P:N	0.031	1.032	0.836–1.274	0.072	1.075		0.87–1.328	
iPTH:L + P:H	0.056	1.058	0.89–1.257	0.104	1.11		0.933–1.32	
iPTH:I + P:H	0.039	1.040	0.895–1.208	0.118	1.126		0.967–1.311	
iPTH:H + P:H	− 0.098	0.907	0.762–1.079	0.000	1		0.838–1.193	

Finally, subgroup analysis further confirmed that patients with aged 65 and over or hypoalbuminemia remained two major risk factors for the development of first episode of peritonitis under different settings of serum PTH/Ca or PTH/P combinations (Fig. 2). There were no significant differences with regard to other factors, such as with or without type 2 DM, gender (male *versus* female), hemoglobin level (Hb ≧ 10 g/dL *versus* Hb < 10 g/dL) across all four categories.

## Discussion

PD-related peritonitis is one of the main patient-oriented outcome measures, linking to technique failure in the future, and continues to be acknowledged as a major barrier for long-term PD utilization from both patients and clinicians’ perspectives^24^. In this study, we provided new evidence showing that deranged mineral bone metabolism with low serum PTH level, in combination with either high Ca level or low/normal P level is associated with higher risk of developing first episode of peritonitis in Taiwanese PD patients, based on a nationwide registry cohort.

Mineral and bone homeostasis has been considerably altered during the progression of CKD, thus mineral bone disease (MBD) becomes a critical issue in advanced CKD patients, including ESKD patients under dialysis^20,22,25^. Clinically, routine examination of serum PTH, Ca and P level have been widely used for monitoring the status of CKD-MBD and for guiding the management accordingly. Recently, aberrant PTH/Ca/P levels have been linked to infection in dialysis patients beyond the scope of CKD-MBD and vascular calcification related cardiovascular morbidity and mortality^19,25^. The underlying mechanism may be associated with systemic immune dysregulation^26,27^. In fact, in vitro experimental data have indicated that immune cell functions could be modulated by different concentrations of PTH, Ca or P, although these functional alterations in uremic patients may not be consistent with in vitro data due to their complex interactions. Here, our data from Taiwanese incident PD patient cohort demonstrated that serum low PTH, high Ca, or low P level at baseline, is associated with worse peritonitis-free survival during 5 -year longitudinal follow up. Interestingly, further analysis of paired combination data (“PTH + Ca”, “PTH + P”, “Ca + P”) revealed that “low PTH + high Ca”, “low PTH + low P”, or “high Ca + low P” has the worst peritonitis-free survival among the nine pairs in each combinational dataset, respectively. These findings hint that PTH/Ca/P are highly interlinked and may have synergistic effects on peritonitis occurrence in this cohort. Notably, after adjustment for age, gender and hypoalbuminemia (serum albumin < 3.7 g/dL *versus* ≧ 3.7 g/dL), we found that low PTH, high Ca, or low P alone becomes no statistical significance in the Cox regression model . Only “low PTH + high Ca” and “low PTH + low (or normal) P” are significantly associated with higher risk of experiencing the first episode of peritonitis. In addition, old age and hypoalbuminemia are two major confounding risk factors in this context and these are consistence with previous study. There are several possible explanations for the present findings. First, low PTH has been shown to reduced phagocytosis capability of polymorphic neutrophils^14^. In addition, low PTH or low P is associated with malnutrition or malnutrition-inflammation complex (MIC) in dialysis patients^28,29^, leading to the increased susceptibility to infections. Finally, the role of Ca on immune cell functions in uremic patients is unclear, although it is widely recognized that intracellular calcium signaling plays a pivotal role in phagocytosis and other immune effector functions^30,31^. Together, these hormonal/mineral abnormalities along with malnutrition would compromise the host defense mechanism in PD patients, from the blood circulation to the peritoneal cavity.

The main strength of this study is using nationwide, registry-based database covering the whole PD population in Taiwan. However, this study was limited by the retrospective nature of the design. Therefore, our observations might have been affected by confounding factors and not be generalizable to PD patients elsewhere. Besides, only a single time point measurement of these biomarkers was used to predict outcomes. Hence, the impact of serial alterations could not be properly evaluated. Finally, lack of detailed medications such as vitamin D analogues might affect the data interpretation as all three parameters would be influenced. In fact, it has been shown that oral active vitamin D intake is associated with decreased risk of peritonitis in PD patients^32^ and serum 25-Hydroxyvitamin D level could predict the peritonitis risk^33^. Future work should take into consideration the effects of Vitamin D analogues as well.

In conclusion, this registry-based observational study has demonstrated for the first time that the combination of low PTH and high Ca or low P could be a potential risk factor for peritonitis occurrence. It remains to be examined whether the reversal of these abnormalities could reduce the risk of peritonitis and the exact mechanism underneath these observations in the future.

## Supplementary Information

Supplementary Information.
