# Author Correction: Aberrant serum parathyroid hormone, calcium, and phosphorus as risk factors for peritonitis in peritoneal dialysis patients

**DOI:** 10.1038/s41598-022-10918-1

**Published:** 2022-04-20

**Authors:** Chia-Te Liao, Cai-Mei Zheng, Yen-Chung Lin, Mei-Yi Wu, Yuh-Feng Lin, Yung-Ho Hsu, Chih-Cheng Hsu, Mai-Szu Wu

**Affiliations:** 1 Division of Nephrology, Department of Internal Medicine, Shuang Ho Hospital, Taipei Medical University, New Taipei City, Taiwan; 2 Division of Nephrology, Department of Internal Medicine, School of Medicine, College of Medicine, Taipei Medical University, Taipei, Taiwan; 3 TMU-Research Center of Urology and Kidney (TMU-RCUK), Taipei Medical University, Taipei, Taiwan; 4 Division of Nephrology, Department of Internal Medicine, Taipei Medical University Hospital, Taipei Medical University, Taipei City, Taiwan; 5 Institute of Epidemiology and Preventive Medicine, College of Public Health, National Taiwan University, Taipei, Taiwan; 6 Center for Health Policy Research and Development, National Health Research Institutes, Miaoli County, Taiwan; 7 Graduate Institute of Clinical Medicine, College of Medicine, Taipei Medical University, Taipei, Taiwan; 8 Division of Nephrology, Department of Medicine, Tri-Service General Hospital, National Defense Medical Center, Taipei, Taiwan

Correction to: *Scientific Reports* https://doi.org/10.1038/s41598-020-80938-2, published online 13 January 2021

The original version of this Article contained an error in Affiliation 2, which was incorrectly given as ‘Department of Internal Medicine, School of Medicine, College of Medicine, Taipei Medical University, Taipei, Taiwan.’

The correct affiliation is listed below:

Division of Nephrology, Department of Internal Medicine, School of Medicine, College of Medicine, Taipei Medical University, Taipei, Taiwan.

The original Article and its accompanying Supplementary Information file have been corrected.

