# Supplementary material for: Aberrant serum parathyroid hormone, calcium, and phosphorus as risk factors for peritonitis in peritoneal dialysis patients
Source: Sci Rep. 2021 Jan 13;11:1171. doi: 10.1038/s41598-020-80938-2 (PMC7806837; doi:10.1038/s41598-020-80938-2)
Supplement: Supplementary file 1 — Supplementary Information. [file 41598_2020_80938_MOESM1_ESM.docx]

**Title page**

**Title: Aberrant serum parathyroid hormone, calcium, and phosphorus as risk factors for peritonitis in peritoneal dialysis patients**

Chia-Te Liao^1,2,3^, Cai-Mei Zheng^1,2,3^, Yen-Chung Lin^2,3,4,^, Mei-Yi Wu^1,2,3,5^, Yuh-Feng Lin^1,2,3,7,8^, Yung-Ho Hsu^1,2,3^, Chih-Cheng Hsu^6^, Mai-Szu Wu^1,2,3^

1. Division of Nephrology, Department of Internal Medicine, Shuang Ho Hospital, Taipei Medical University, Taipei, Taiwan
2. Division of Nephrology, Department of Internal Medicine, School of Medicine, College of Medicine, Taipei Medical University, Taipei, Taiwan
3. TMU-Research Center of Urology and Kidney (TMU-RCUK), Taipei Medical University, Taipei, Taiwan
4. Division of Nephrology, Department of Internal Medicine, Taipei Medical University Hospital, Taipei Medical University, Taipei, Taiwan
5. Institute of Epidemiology and Preventive Medicine, College of Public Health, National Taiwan University, Taipei, Taiwan
6. Center for Health Policy Research and Development, National Health Research Institutes, Miaoli County, Taiwan
7. Graduate Institute of Clinical Medicine, College of Medicine, Taipei Medical University, Taipei, Taiwan
8. Division of Nephrology, Department of Medicine, Tri-Service General Hospital, National Defense Medical Center, Taipei, Taiwan

**Correspondence to:**

Dr. Yen-Chung Lin, M.D., Ph.D.

Division of Nephrology

Department of Internal Medicine

Taipei Medical University Hospital

Taipei City, Taiwan

Email: [yclin0229@tmu.edu.tw](about:blank)

Dr. Mai-Szu Wu, M.D.

Division of Nephrology

Department of Internal Medicine

Taipei Medical University Shuang Ho Hospital

New Taipei City, Taiwan

Email: [maiszuwu@gmail.com](about:blank)

**Supplemental Figure 1: Flowchart of the whole population**


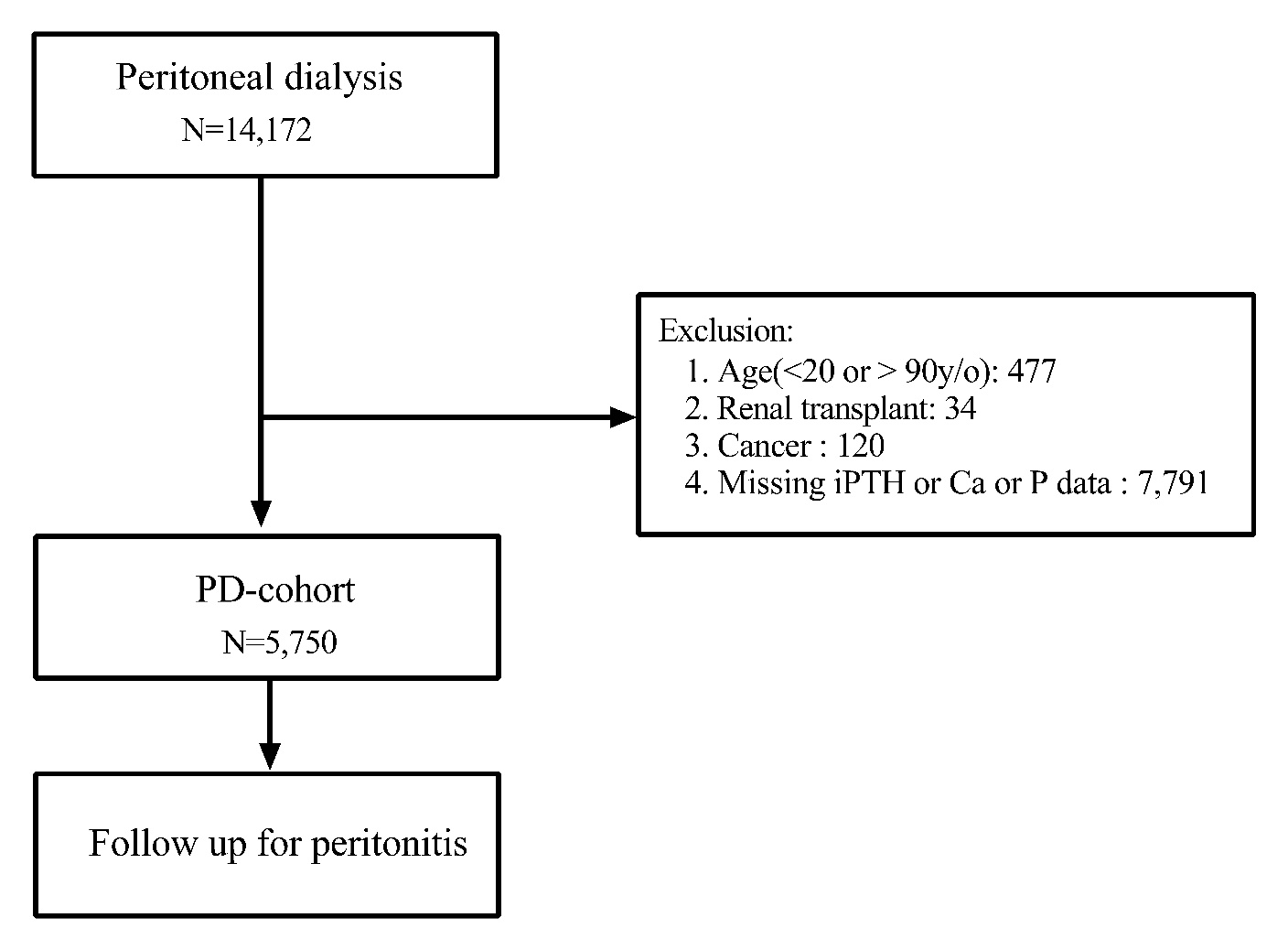


**Supplemental Figure 2: Peritonitis-free survival in different categories according to the calcium/phosphate/calcium plus phosphate levels**


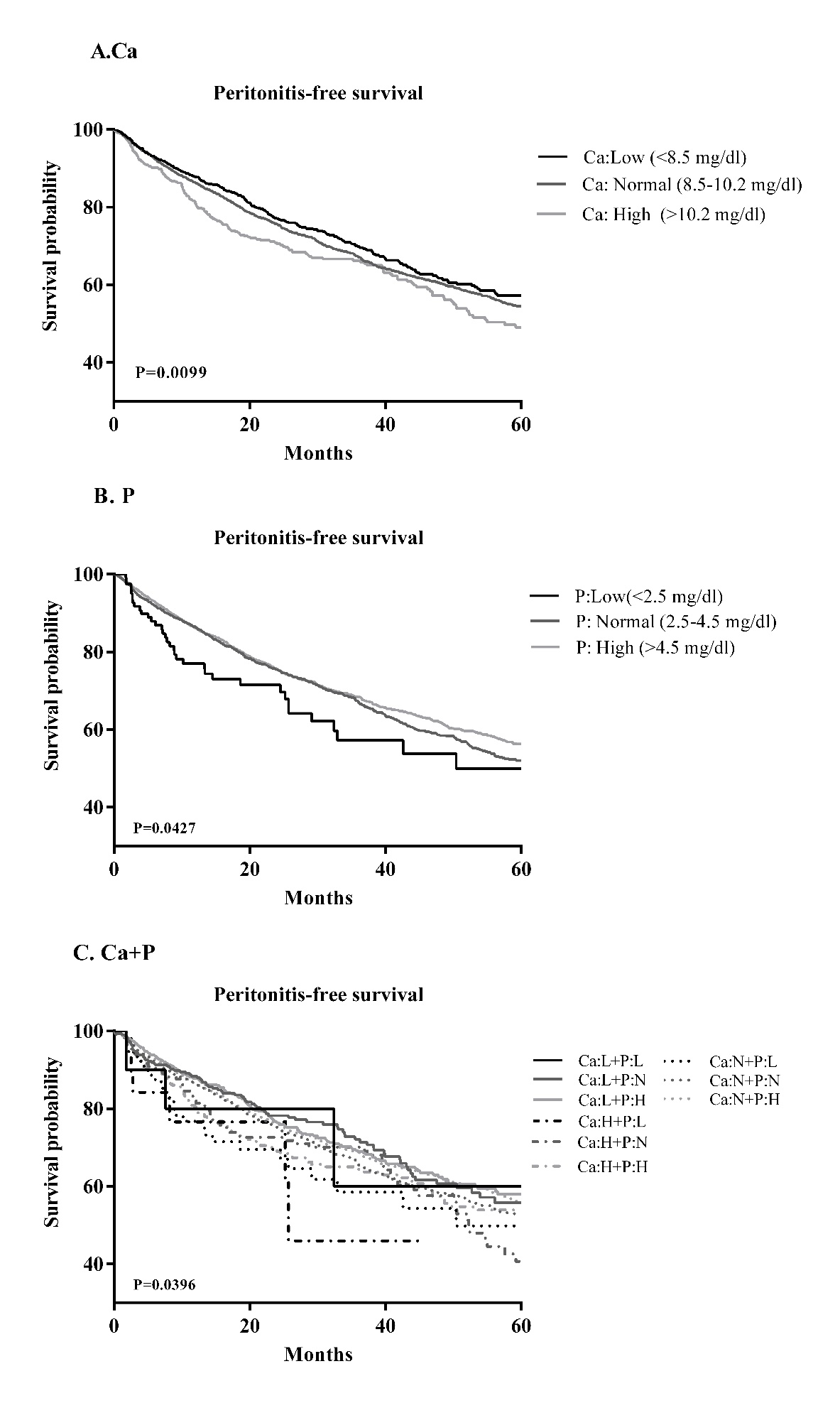


**Supplemental tables**

**
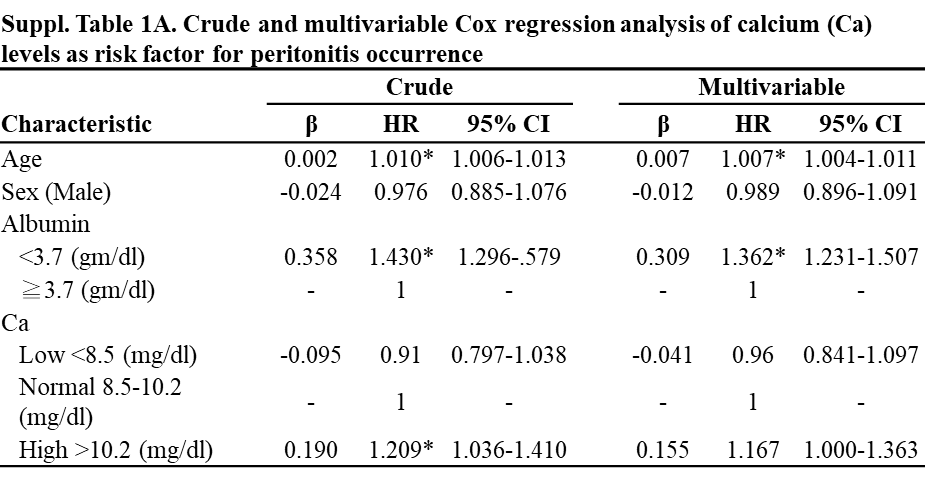
**

**
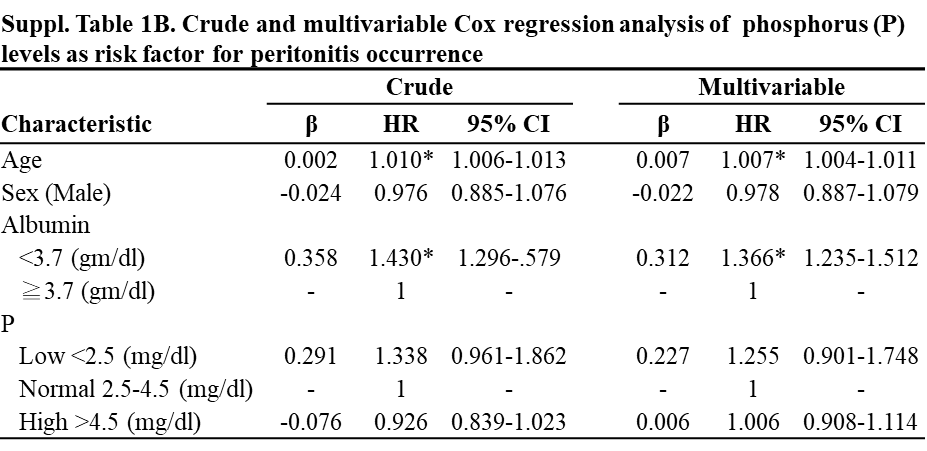
**

**
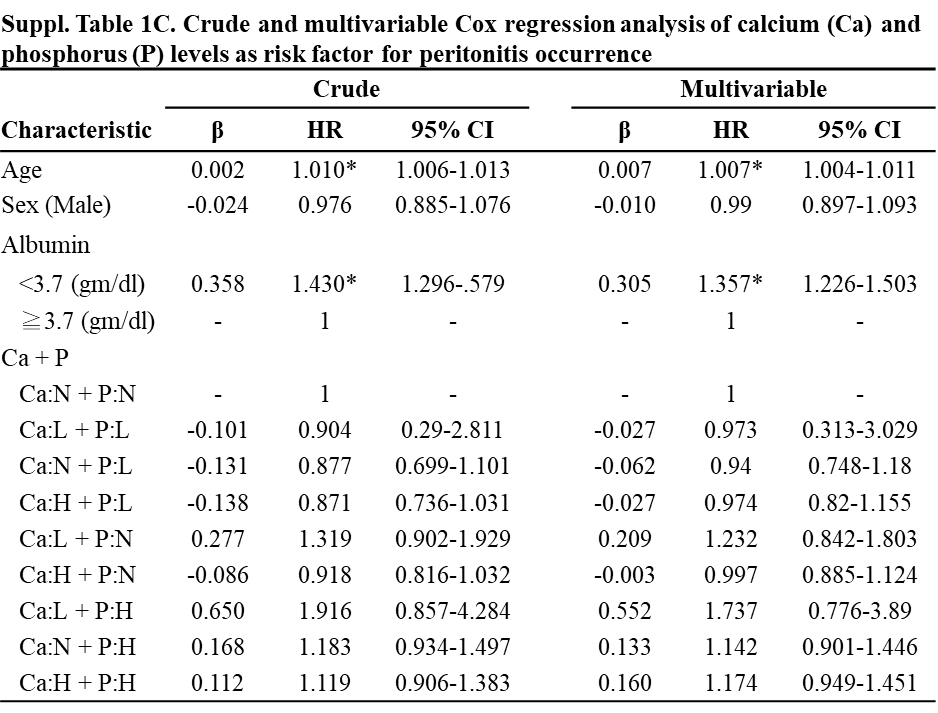
**
